# Polymer Matrix and Manufacturing Methods in Solid Dispersion System for Enhancing Andrographolide Solubility and Absorption: A Systematic Review

**DOI:** 10.3390/pharmaceutics16050688

**Published:** 2024-05-20

**Authors:** Pratchaya Tipduangta, Sunee Chansakaow, Pimpimon Tansakul, Rungarun Meungjai, Piyameth Dilokthornsakul

**Affiliations:** 1 Department of Pharmaceutical Sciences, Faculty of Pharmacy, Chiang Mai University, Chiang Mai 50200, Thailand; pratchaya.t@cmu.ac.th (P.T.); sunee.c@cmu.ac.th (S.C.); rungarun_me@cmu.ac.th (R.M.); 2 The College of Herbal Pharmacy of Thailand, The Pharmacy Council of Thailand, Nonthaburi 11000, Thailand; 3 Department of Pharmacognosy and Pharmaceutical Botany, Faculty of Pharmaceutical Sciences, Prince of Songkla University, Songkhla 90110, Thailand; pimpimon.t@psu.ac.th; 4 Center for Medical and Health Technology Assessment (CM-HTA), Department of Pharmaceutical Care, Faculty of Pharmacy, Chiang Mai University, Chiang Mai 50200, Thailand

**Keywords:** *Andrographis paniculata*, andrographolide, solid dispersion, aqueous solubility, absorption

## Abstract

**Background:** Andrographolide (ADG) has poor aqueous solubility and low bioavailability. This study systematically reviews the use of solid dispersion (SD) techniques to enhance the solubility and absorption of ADG, with a focus on the methods and polymers utilized. **Methodology:** We searched electronic databases including PubMed, Web of Science, Scopus^®^, Embase and ScienceDirect Elsevier^®^ up to November 2023 for studies on the solubility or absorption of ADG in SD formulations. Two reviewers independently reviewed the retrieved articles and extracted data using a standardized form and synthesized the data qualitatively. **Results:** SD significantly improved ADG solubility with up to a 4.7-fold increase and resulted in a decrease in 50% release time (T_1/2_) to less than 5 min. SD could also improve ADG absorption, as evidenced by higher C_max_ and AUC and reduced T_max_. Notably, Soluplus-based SDs showed marked solubility and absorption enhancements. Among the five SD techniques (rotary evaporation, spray drying, hot-melt extrusion, freeze drying and vacuum drying) examined, spray drying emerged as the most effective, enabling a one-step process without the need for post-milling. **Conclusions:** SD techniques, particularly using Soluplus and spray drying, effectively enhance the solubility and absorption of ADG. This insight is vital for the future development of ADG-SD matrices.

## 1. Introduction

*Andrographis paniculata* is an herbal medicine commonly used in traditional medicine across Asia. In Thailand, it has been included in the National List of Essential Herbel Medicines since 2013 [1]. It can be used as monotherapy in the form of dried leaf powder in capsules for pills for sore throats, colds and non-infectious diarrhea. Recently, it was used in treating COVID-19 symptoms during the early COVID-19 pandemic period. Evidence shows that it could inhibit viral division without cytotoxic effects [2]. The recommended dosage for sore throat and cold is 1.5–3 g four times a day, while the dosage of 500 mg–2 g four times a day is for diarrhea via oral route in tablets or capsules [3], resulting in a bulky administration dose and decreasing patients’ compliance.

Andrographolide (ADG), a diterpene lactone with pharmacological effects such as anti-inflammatory, antiviral, antibacterial, antioxidant and immune-boosting properties, serves as a marker to control the specification of *Andrographis paniculata* extract and its products [3,4]. However, it exhibits poor aqueous solubility (3.29 ± 0.73 µg/mL at 25 °C) with a log *p* value of 2.632 ± 0.135, and its crystalline form has a melting point at 243 °C. The molecular weight of ADG is 350.45 g·mol^−1^ [5,6]. Ye et al. reported low oral absorption of ADG (dose 120 mg/kg) with a C_max_ of 0.23 ± 0.05 µg/mL in rats [7]. ADG undergoes rapid metabolism in the biological condition as 14-deoxy-12-sulfo- andrographolide and is also subject to efflux by P-glycoprotein at the ileum and colon sites, limiting its bioavailability to only 2.67% [7]. The combination of poor aqueous solubility and low bioavailability results in an increase in the bulkiness of ADG dosage forms and frequency of administration, leading to decreased patient compliance [8,9,10].

To address the low solubility and bioavailability, various pharmaceutical techniques such as microemulsion and solid dispersion (SD) have been explored. The SD technique is frequently employed to increase the aqueous solubility of poorly water-soluble active pharmaceutical ingredients due to its robustness and feasibility for industrial-scale manufacturing, with numerous original medicines and generic drugs adopting this technique [11,12]. SD has been used for enhancing aqueous solubility by converting active pharmaceutical ingredients into their amorphous form where the molecules have higher energy, facilitating water solubility [13]. New generations of SD act as solubilizers to increase aqueous solubility, such as Soluplus, which includes a surfactant function that can form micelles and increase the solubility of poorly water-soluble drugs [14]. Additionally, the hydrophilic polymer can aid in wetting, making the drug easier to dissolve. SD is in solid dosage form, which is easier to manufacture into tablets or capsules compared to nano- and microemulsions, which are in liquid form. Thus, SD technology has been used and evidenced in increasing the aqueous solubility and bioavailability of ADGs in several studies [15,16,17,18].

There are several factors, such as preparation techniques, types of polymers, ADG content and particle size, that could affect ADG SD efficacy, resulting in various ranges of ADG aqueous solubility. Several review articles related to ADG formulations have been published, but they mainly focus on formulations overall rather than specifically on SD and its factors. To the best of the authors’ knowledge, there is no summative evidence showing the effect of different SD preparation techniques, polymer use, ADG content or particle size on the aqueous solubility and absorption of ADG. This information is essential for deciding the appropriate ADG formulation and preparation process, which could be used to consider scaling up for international industrial use for medical use. Therefore, this systematic review aims to summarize the essential information on the effect of different SDs of ADG on aqueous solubility and absorption and provide a discussion on SD ADG formulations and their manufacturing process.

## 2. Materials and Methods

### 2.1. Search Strategy

To identify studies determining the effect of different SDs of ADG on aqueous solubility and absorption, PubMed, Web of Science, Scopus^®^, Embase and ScienceDirect were searched from their inception to November 2023. Search terms included andrographolide and solid dispersion. Our search strategies are presented in Supplementary Materials. The study was conducted according to PRISMA guidelines, and the study protocol was registered at PROSPERO (CRD42022303337).

### 2.2. Study Selection

All in vitro and in vivo studies reporting the effect of SD of ADG on aqueous solubility and absorption were eligible for our systematic review. The inclusion criteria were (1) studies evaluating the effect of SD of ADG on aqueous solubility, (2) studies conducted on in vitro or in vivo models and (3) studies reported in the English language. All titles and abstracts of retrieved articles were independently reviewed by PT and RM. All disagreements were solved by a discussion with PD or SN.

### 2.3. Data Extraction and Data Analysis

A standardized data extraction form was created in Microsoft Excel^®^ for Microsoft 365 MSO (Version 2404 Build 16.0.17531.20140) 64-bit. The extracted data for in vitro studies were type of study, country of study, type of SD preparation methods, polymers matrices, %ADG, particle size, dissolution medium, maximum ADG release and time to half release (T_1/2_), while the extracted data for in vivo studies were polymer matrices, study design, type of animal model, characteristics of animals, maximum concentration (C_max_), time to maximum concentration (T_max_) and area under the curve (AUC). The data extraction was performed by PT and RM. All disagreements were solved by consensus. Data were summarized qualitatively and classified by polymer matrix and SD preparation technique. The polymer matrix was used to classify SD of AGD because it is at the core of SD, serving as the medium in which drug molecules are dispersed [19], which might affect the ability of aqueous solubility. The SD preparation technique was also used to classify the SD of AGD because it is a crucial factor in obtaining a homogeneous SD matrix.

## 3. Results

### 3.1. Study Characteristics

**Figure 1:**
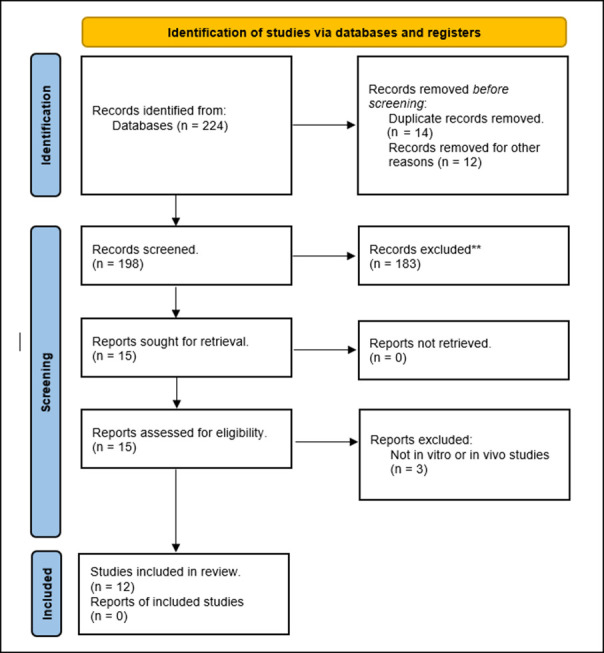
PRISMA flow diagram. ** Do not meet the inclusion criteria.

A total of 224 articles were retrieved from our database searches. Of those, 14 articles were duplicated, and 12 articles were non-English articles. Thus, 198 articles were screened. Of those, 15 articles were eligible for full-text review, and only 12 articles were included in our systematic review. The three articles excluded in the full-text review process were studies not reporting aqueous solubility or absorption (Figure 1).

### 3.2. In Vitro Release Studies

Eleven in vitro studies [5,6,10,15,17,18,20,21,22,23,24] were conducted to assess the SD of ADG aqueous solubility. They could be classified according to polymer matrices into eight groups, namely polyethylene glycol (PEG), the polyvinyl carprolactam–polyvinyl acetate–polyethylene glycol graft copolymer (PCL-PVAc-PEG; Soluplus), polyvinylpyrrolidone (PVP), PVP with vinyl acetate (PVP-VA), hydroxypropyl methylcellulose (Hypromellose; HPMC), silicon dioxide, chitosan and Poloxamer 188. When classifying by the SD preparation techniques, a total of five SD preparation techniques were found, namely rotary evaporation, spray drying, hot-melt extrusion, freeze drying and vacuum drying (Table 1). Of those, four SD preparation techniques, namely rotary evaporation, spray drying, freeze drying and vacuum drying, were solvent-based methods, while only hot-melt extrusion was a thermal-based method.

#### 3.2.1. Polymer Matrices

##### PEG

PEG 4000–8000 and their derivatives, such as PEG palmitate, PEG laurate and PEG behenate, were the most common matrices in the formulation of SDs of ADG [6,17,21,24]. A total of twenty-five ADG formulations were prepared using PEG and its derivatives as matrices.

One study indicated that PEG 6000 did not lead to an improvement in ADG solubility compared to the control [17]. However, three other studies showed that PEG could increase ADG aqueous solubility by 2.2 to 4.5 times compared to the control group [6,21,24]. The T_1/2_ of SD of ADG was consistently lower than a control. We observed that a shorter T_1/2_ often was correlated with a higher improvement in aqueous solubility (Table 2).

##### Soluplus

Thirteen formulations from three studies employed Soluplus as the matrix for SD of ADG [17,20,21]. In general, Soluplus could significantly enhance ADG solubility by 1.8- to 4.7-fold compared to controls.

SD using Soluplus could reduce the T_1/2_ of ADG from >120 min in controls to <10 min in most formulations. Notably, there were two formulations for which even when ADG solubility was improved by >3.0-fold, the T_1/2_ remained within the range of 30 to 45 min, which was greater than that observed in other formulations [21] (Table 2).

##### PVP

Two studies with a total of 15 formulations used PVP as the matrix for ADG dispersion [5,18]. In general, SD by PVP could significantly increase ADG aqueous solubility, ranging from 1.8- to 3.2-fold over that of the controls. The T_1/2_ was reduced from >120 min in controls to <10 min in most SD formulations using PVP. It should be noted that three formulations exhibited slightly higher T_1/2_ values of 12.4, 15.2 and 22.5 min [5,18]. This slight increase in the T_1/2_ may be attributed to the higher content of ADG in these formulations, which consisted of 33.33% and 50% ADG contents (Table 2).

##### PVP-VA

Seven formulations in two studies employed PVP-VA in a 6:4 mass ratio (called PVP-VA 64) as the matrix for SD of ADG [17,20]. Overall, PVP-VA 64 significantly improved ADG solubility, which ranged from 2.1- to 3.3-fold higher than that of crystalline ADG. All the formulations exhibited a consistent trend of reducing T_1/2_ to <5 min (Table 2).

##### HPMC

We found only one study with four formulations that used HPMC as the sole matrix to enhance ADG solubility [15]. The results of the study indicated that HPMC, when used as a matrix, could significantly improve ADG solubility, reaching a maximum increase of 2.9-fold with a 15% loading. However, a notable decrease in solubility improvement was observed when ADG loading exceeded 50% [15] (Table 2).

##### Silicon Dioxide

One study used silicon dioxide to formulate an SD of ADG. The study loaded ADG into silicon dioxide in ratios ranging from 1:1 to 1:8, yielding five different formulations with varying ADG contents. These formulations enhanced ADG solubility by 1.2 to 1.7 times and reduced the T_1/2_ to <5 min in the formulations with an ADG content of 11.11% [23]. The study highlighted a trend of an inverse relationship of ADG loading in silicon dioxide with solubility and T_1/2_ enhancement (Table 2).

##### Chitosan

A study by Sari et al. employed Chitosan as an SD matrix in three formulations with ADG contents ranging from 16% to 25% [22]. They found that chitosan enhanced ADG solubility by approximately 1.5 times, with a T_1/2_ of 4 and 13 min, a significant reduction compared to the control’s 106 min [22] (Table 2).

##### Poloxamer 188

A study by Song et al. used Poloxamer 188 as an SD matrix to enhance ADG solubility. However, the maximum ADG release did not show any superiority of Poloxamer 188 compared to the control. However, SD using Poloxamer 188 showed a faster dissolution in the early-release study [17] (Table 2).

#### 3.2.2. SD Preparation Techniques

##### Rotary Evaporation

Rotary evaporation was found to be the most common technique among the included studies for SD preparation. It was employed in 35 formulations of four studies [18,20,21,23]. The ADG solubility improvement achieved with the SD prepared using the rotary evaporation technique ranged from 1.1- to 3.8-fold higher than that of the original form. Twenty-one formulations exhibited an ADG solubility improvement within the range of 2.0- to 3.0-fold. The T_1/2_ varied widely, spanning from 2.73 to 54 min. Twenty out of thirty-five formulations had a T_1/2_ < 10 min (Table 2).

##### Spray Drying

Eighteen formulations from four studies employed the spray drying method [5,15,22,24]. It demonstrated the highest ADG solubility improvement, with an impressive 4.5-fold enhancement compared to other techniques. However, ADG solubility improvement varied from 1.4- to 4.5-fold. The overall trend of T_1/2_ indicated a rapid release of ADG. Thirteen formulations had a T_1/2_ of <10 min. The fastest T_1/2_ was as short as 2 min. However, two formulations were reported to have a T_1/2_ >30 min [15]. This might be attributed to the high ADG loading (75–85%), resulting in a delayed T_1/2_ (Table 2).

##### Hot-Melt Extrusion

Nine formulations from two studies employed hot-melt extrusion to prepare ADG solid dispersions [17,20]. Most of the formulations exhibited a solubility enhancement of approximately 2.5- to 3.0-fold, with the maximum being 4.7-fold and the minimum being 1.0-fold. Eight of nine formulations demonstrated a T_1/2_ of <5 min, indicating a rapid release of ADG. However, one formulation had a T_1/2_ exceeding 120 min. Notably, this formulation also had solubility equal to that of its control (Table 2).

##### Freeze Drying

Zeng et al.’s study is the only one that used a freeze drying technique to prepare two SD of ADG formulations [6]. These formulations substantially improved ADG solubility, with the enhancements of 3.3-fold and 4.5-fold, while their T_1/2_ values were 7.43 min and 8.96 min, respectively (Table 2).

##### Vacuum Drying

Zhao et al. employed the vacuum drying technique to prepare eight SD of ADG formulations [24]. These formulations significantly improved ADG solubility, with the enhancements ranging from 3.0- to 4.2-fold. The overall T_1/2_ of the vacuum drying technique was higher than that of other techniques. The T_1/2_ of this technique was on average 25 min, while the T_1/2_ values of other techniques were <10 min. The T_1/2_ of the SD using the vacuum drying technique varied from 7.7 to 43.8 min (Table 2).

### 3.3. In Vivo Pharmacokinetic Study

A total of nine formulations with seven different types of polymers were examined in pharmacokinetic studies involving SD of ADG [10,15,17,18,21]. These formulations were selected based on screening results obtained from in vitro release studies. The research employed various animal models, such as Wistar rats, Beagle dogs, Albino rats and Sprague Dawley rats. In these studies, SDs of ADG were administered in a single dose, and blood samples were collected from the animals over a period of 8–36 h. The details of the in vivo pharmacokinetic studies are reported in Table 3.

#### 3.3.1. PEG

Song et al. employed PEG 6000 as an SD matrix of ADG [17]. They observed the improvements in the pharmacokinetic parameters of absorption. Specifically, there was a slight increase in C_max_ and AUC compared to a control. The T_max_ also showed a slight increase, which can be attributed to the previous parameters (Table 3).

#### 3.3.2. Soluplus

Soluplus was employed as the SD matrix of ADG in two studies. The studies demonstrated a consistent trend of higher C_max_ and AUC compared to controls. Specifically, the AUC of ADG was improved by 3.2-fold in a study by Song et al. and 1.18-fold in a study by Nitave et al. [16,21]. Soluplus exhibited outstanding enhancement in ADG absorption compared to other polymers, as observed in a study by Song et al. [17] (Table 3).

#### 3.3.3. PVP

Ma et al. utilized a combination of PVP with polyethoxylated castor oil. The pharmacokinetics of SD of ADG using the PVP combination matrix slightly increased in C_max_ and AUC. A lower T_max_ by one-fifth was observed [18]. The finding indicated the rapid absorption of the SD of ADG using PVP with polyethoxylated castor oil (Table 3).

#### 3.3.4. PVP-VA

PVP-VA 64 was solely used by Song et al. to enhance the in vivo absorption of ADG. The C_max_ and AUC of the SD of ADG using PVP-VA 64 were notably increased compared to the control (1.75-fold increase in AUC) [17]. The findings indicated an absorption enhancement with the SD of ADG using PVP-VA 64.

#### 3.3.5. HPMC

Three formulations used HPMC from two studies, with one of the formulations being a combination of HPMC, lactose and sodium carboxymethyl starch at a ratio of 3:2:3. A study by Ma et al. observed that both the sole HPMC formulation and the combined HPMC formulation significantly improved ADG absorption, as shown by a higher C_max_ and AUC. It was noted that AUC was enhanced by 4.12- and 4.73-fold compared to the control for sole HPMC and combined HPMC, respectively [15]. In another study, the AUC improvement of an SD of ADG compared to control was not calculated because the control was commercial ADG dripping pills. Nevertheless, the SD of ADG using HPMC had a higher C_max_, a higher AUC and a lower T_max_, indicating faster absorption [10] (Table 3). However, it is important to note that the studies did not mention the types and grades of HPMC. Different types and grades of HPMC might affect the characteristics and effectiveness of SD of ADG.

#### 3.3.6. Poloxamer

Poloxamer 188 was used by Song et al. as an SD matrix to enhance ADG absorption [17]. The findings indicated a slight increase in C_max_ and T_max_ and a 1.25-fold AUC improvement compared to the control (Table 3).

## 4. Discussion

### 4.1. Optimizing SD of ADG Formulation

Twelve included articles stand on common ground in indicating that the SD technique can improve the aqueous solubility of ADG. Different polymers demonstrated a wide range of ADG solubility improvements. We found that Soluplus is the most promising polymer for SD of ADG. Consistent evidence supports its efficacy in both in vitro solubility and in vivo pharmacokinetic studies. This can be attributed to its ability to stabilize ADG in an amorphous form and its solubilizing effect, forming micelles that increase the solubility of this poorly water-soluble compound [25]. In addition, Soluplus could effectively reduce T_max_. The reduction in T_max_ of Soluplus is attributed to the inhibition of P-glycoprotein efflux at the duodenum [26].

PEG and its derivatives exhibit the highest in vitro ADG solubility improvement among the polymers, but there is only one available in vivo pharmacokinetic study. Its results are inconsistent with in vitro solubility studies. It also indicates that PEG is a promising polymer for SD of ADG; however, more in vivo pharmacokinetic studies are required to improve credibility of the findings.

HPMC and PVP-VA 64 also show consistent results in both in vitro solubility studies and in vivo pharmacokinetic studies, but they have fewer reported formulations compared to Soluplus and PEG. HPMC and PVP-VA 64 have about 0–2 times the solubility compared to Soluplus and PEG. Thus, they might be options when Soluplus or PEG is not feasible.

There is limited evidence on the use of silica dioxide and chitosan for SD of ADG. Evidence of silica dioxide and chitosan is available only in in vitro solubility studies. No in vivo pharmacokinetic studies were found. Thus, there is a limited application of silica dioxide and chitosan for SD of ADG.

Besides polymer selection, active compound content is a critical factor affecting the solubility improvement of SD formulations. Excessive loading can lead to recrystallization, minimizing aqueous solubility, while low loading tends to show better stability but results in a bulkier formulation. Balancing active compound loading within the stability safety zone is indeed an art of formulations. We found that the range of ADG loading in SD falls to 11–85%. The states of ADG were interpreted from X-ray diffraction reported in studies. ADG in a PEG matrix tends to be partially amorphous. This might be because PEG’s semi-crystalline nature promotes heterogeneous nucleation and induces ADG crystallization, leading to partially amorphous and crystalline ADG [27]. Zhao et al. reported that the carbon chain length of PEG fatty acid ester can improve the space-limiting effect of the carrier material on the ADG molecule [28]. A longer carbon chain length of PEG was not better for limiting the molecular mobility of ADG and improving the thermodynamic stability [28]. Even though ADG was in a partially amorphous state, the improvement in dissolution compared to ADG in the same study was not notably low compared to other polymers. However, a firm conclusion regarding the effect of partially amorphous ADG on in vivo kinetic studies cannot be drawn due to insufficient evidence. On the other hand, PVP-K30, PVP-VA and Soluplus demonstrated that ADG was in a fully amorphous state. Our included studies suggest that amorphous polymers with ADG loading below 33% achieve a fully amorphous state of ADG [5,16,17,18,20]. Regarding ADG SD stability, Gou et al. reported that ADG SDs prepared with Soluplus and PVP-VA64 were chemically stable and unchanged in their dissolution after expose to the stress conditions (60 °C with 0% RH and room temperature with 75 or 92.5% RH for 7 days) [20]. Zhang et al. reported a slight decrease in ADG content and a cumulative dissolution of approximately 3% after storage in a stressed condition (40 °C and 75% RH) for 3 months of SD with silicon dioxide as a matrix [23]. Lomlim et al. reported that AGD PVP K-30 SD was less stable compared to its crystalline form under stressed conditions (45, 60 and 70 °C with 75% RH) for 84 days [29]. These studies served as examples for predicting the stability trend of ADG in SD. Since long-term stability data of ADG are not available and the theoretical glass transition of ADG SD formulations cannot be estimated due to a lack of ADG glass transition data, a low ADG loading is preferable to ensure the complete amorphization of ADG during product shelf-life. Therefore, an ADG loading ratio below 25% is preferable, and stability studies of ADG SDs that comply with the ICH Q1A guideline are required to ensure the product shelf-life [30].

Particle size is also an important factor in aqueous solubility. Smaller particle sizes could enhance the solubility of the drug substance. In the context of SD, the particle size of the active substance-loaded matrices exhibits an effect on solubility. Interestingly, we found that only three studies reported the particle size of their SD formulations, and the particle size fell in the range of 2–150 µm [5,10,24]. Notably, small particles were obtained through wet milling [10] and the spray drying technique [5]. For drug manufacturers, strict control over the particle size of SD matrices is essential to minimize deviations in flowability and dissolution of the products. Therefore, ensuring that the particle size of SD of ADG matrices is within a validated range should be considered a critical quality attribute in the manufacturing process. This attention to particle size contributes to the overall efficacy and performance of the solid dispersion formulation.

### 4.2. Feasible SD of ADG Preparation Process

Rotary evaporation was found to be the most common method for preparing SDs of ADG due to its simplicity and shared equipment with solvent concentration techniques. In contrast, spray drying, vacuum drying, and hot-melt extrusion require specific equipment, making their lab-scale implementation more expensive than a rotary evaporator. The rotary evaporation technique is frequently employed to investigate the physicochemical compatibility and stability of active pharmaceutical ingredients and polymer matrices on a laboratory scale [31,32,33]. The use of an SD matrix prepared by rotary evaporation necessitates post-milling and sieving to achieve a consistent particle size.

Vacuum drying and freeze drying provides several advantages by employing low temperatures in the drying process. They minimize the risk of drug degradation from oxidation. However, they have limitations requiring a post-milling and sieving process to control particle sizes. Freeze drying involves dissolving or dispersing the active compound and polymer in aqueous solutions. While organic solvents are prohibited for the freeze drying process, vacuum drying is feasible for their use. The freeze drying process, involving freezing, primary heating and secondary heating, requires an extended operating time. Freeze drying might not be the preferred option for industrial-scale SD methods for poorly water-soluble substances such as ADG.

Spray drying and hot-melt extrusion are frequently utilized in industrial-scale SD productions due to their robustness and continuous processes. Various commercial SD products were prepared by these two methods [12,32]. Spray drying offers the advantage of obtaining high-surface-area particles without the need for post-milling, distinguishing it from hot-melt extrusion. In addition, Zhao et al. reported that the spray drying method significantly reduced the crystallinity of ADG compared to the vacuum drying method [24]. This results from the fast solvent evaporation of the spray drying method, which enhances ADG’s amorphous state in the SD formulations. In contrast, HPMC and chitosan were prepared by the spray drying technique but did not achieve fully amorphous ADG due to their preparation techniques, which disperse ADG in water or 0.3% acetic acid in which ADG does not dissolve but the polymer does [15,22]. Thus, ADG and the polymers were not homogeneously solidified by spray drying. Spray drying has a limitation on the energy efficiency during its particle solidification process [34]. Pharmaceutical spray dryers and hot-melt extruders are dedicated process equipment for preparing solid dispersions.

### 4.3. Knowledge Generated from Our Findings

A recent systematic review study on trends in advanced oral drug delivery systems for curcumin indicates that SD technology could improve in vivo absorption of curcumin in one human study and eleven animal studies [35]. From our study, we can draw a firm and consistent conclusion that the use of SD technology is a promising technique to enhance aqueous solubility and in vivo absorption of ADG. We could also add more important information that polymer matrices can be ranked as Soluplus > PEG derivatives > HPMC > PVP-VA64 > Poloxamer 188 > silicon dioxide > chitosan. Soluplus is the most appropriate SD matrix for AGD, demonstrating a significantly increased ADG aqueous solubility while concurrently inhibiting P-glycoprotein efflux, thereby enhancing ADG blood concentration and leading to heightened ADG bioavailability. Spray drying is the most suitable technique for preparing ADG SDs. Our findings could be used as important information for product registration. They could help manufacturers outline the advantages and disadvantages of manufacturing methods and select suitable manufacturing methods for producing SDs of ADG. They might be a part of the information in Section 3.2.P.2 (Pharmaceutical Development) for drug product registration as per the ICH Q8 guidelines for SD of ADG. Nevertheless, the number of SD of ADG pharmacokinetics studies is limited; future pharmacokinetic studies are warranted to validate the characteristics and efficacy of SD of ADG.

## 5. Conclusions

This systematic review provides updated important information on the appropriate polymer matrices and SD preparation techniques for SD of ADG. SD could improve the aqueous solubility and absorption of ADG. The potential polymer matrices for SD preparation are PEG, Soluplus, PVP, PVP-VA 64, HPMC and Poloxamer 188. However, silicon dioxide and chitosan might be considered. Rotary evaporation is a common SD preparation technique for formulating SDs of ADG; however, it might not be suitable for manufacturing. The spray drying technique is an appropriate alternative for formulating SDs of ADG on the manufacturing scale, with relatively strong evidence of its effectiveness. Our findings are important pieces of evidence for future research and development of SDs of ADG on the manufacturing scale.

## Figures and Tables

**Table 1:** Study design of andrographolide solid dispersions, their preparation methods, polymer matrices, and particle size.

Authors	Year	Country	In Vitro Release Study	In Vivo Absorption Study	Category of Solid Dispersion Preparation Method	Polymer Matrices	Particle Size (µm)	References
Song et al.	2020	China	✓	✓	Thermal Base	Polyethylene glycol 6000	NR	[17]
						Polyvinylpyrrolidone VA64		
						Poloxamer 188		
						Soluplus		
Yen et al.	2020	Taiwan	✓	✓	Solvent Base	PEG 6000	NR	[18]
						PVP K15		
						PVP K30		
						PVP K90		
						PVP K30, Tween 80		
						PVP K30, Kolliphor EL		
Zeng et al.	2020	China	✓	✕	Solvent Base	PEG4000 palmitate	NR	[6]
						PEG8000 palmitate		
Gou et al.	2019	China	✓	✕	Thermal Base	Kollidon VA64	NR	[20]
						Soluplus		
					Solvent Base	Kollidon VA64		
						Soluplus		
Sari et al.	2019	Indonesia	✓	✕	Solvent Base	Chitosan	NR	[22]
Zhao et al.	2019	China	✓	✕	Solvent Base	PEG4000	42.8 (d0.5)	[24]
						PEG4000 laurate	52.7	
						PEG4000 palmitate	79.6	
						PEG4000 behenate	64.3	
						PEG8000	33.1	
						PEG8000 laurate	48.6	
						PEG8000 palmitate	52.4	
						PEG8000 behemate	48.5	
					Solvent Base	PEG4000	109.4	
						PEG4000 laurate	131.3	
						PEG4000 palmitate	96	
						PEG4000 behenate	125.4	
						PEG8000	113.3	
						PEG8000 laurate	127.2	
						PEG8000 palmitate	99.8	
						PEG8000 behemate	144.7	
Ma et al.	2018	China	✓	✓	Solvent Base	15% HPMC	NR	[15]
					Solvent Base	25% HPMC		
					Solvent Base	15% HPMC with 15% Lactose, 15% SCS		
Nitave et al.	2018	India	✓	✕	Solvent Base	Soluplus	NR	[21]
						PEG 6000	NR	
Nitave et al.	2018	India	✕	✓	NR	Soluplus	NR	[16]
Zhang et al.	2016	China	✓	✕	Solvent Base	Silicon dioxide	NR	[23]
					Solvent Base	Silicon dioxide	NR	
Zhang et al.	2015	China	✓	✓	Solvent Base	HPMC	3.17 (mean size)	[10]
Bothiraja et al.	2009	India	✓	✕	Solvent Base	Polyvinylpyrrolidone (PVP K-30)	3.6 ± 0.3	[5]
							3.1 ± 0.6	
							2.8 ± 0.4	

**Abbreviations:** NR: not reported. ✓ refer to available and ✕ refer to not available.

**Table 2:** In vitro release study of andrographolide solid dispersion formulas.

Polymer Matrix	Solid Dispersion Preparation Method	Dissolution Medium	% Andrographolide Loading	Andrographolide State in SD	Maximum Andrographolide Release (%)	Dissolution Improvement Compared to Andrographolide in the Same Study (Fold)	T_1/2_ (min)	References
PEG 6000	Hot-Melt Extrusion	Phosphate buffer pH 6.8	12.5%	NR	18.5	1.0	>120	[17]
PEG 4000	Vacuum Drying	Distilled water	25%	Crystalline	66.7 ± 1.5	3.0	43.8	[24]
PEG 4000 laurate	Vacuum Drying	Distilled water	25%	Crystalline	73.6	3.3	7.7	
PEG 4000 palmitate	Vacuum Drying	Distilled water	25%	NR	82.1	3.7	13.1	
PEG 4000 behenate	Vacuum Drying	Distilled water	25%	NR	78.5	3.5	8.6	
PEG 4000	Spray Drying	Distilled water	25%	Partial amorphous	89.2 ± 1.3	4.0	4.2	
PEG 4000 laurate	Spray Drying	Distilled water	25%	Partial amorphous	98.7	4.4	2.5	
PEG 4000 palmitate	Spray Drying	Distilled water	25%	NR	101	4.5	2.7	
PEG 4000 behenate	Spray Drying	Distilled water	25%	NR	96.5	4.3	2.6	
PEG 8000	Vacuum Drying	Distilled water	25%	NR	74.2 ± 1.8	3.3	29.9	
PEG 8000 laurate	Vacuum Drying	Distilled water	25%	NR	74.4	3.3	37.8	
PEG 8000 palmitate	Vacuum Drying	Distilled water	25%	NR	93.2	3.3	28.9	
PEG 8000 behenate	Vacuum Drying	Distilled water	25%	Partial amorphous	73.4 ± 1.6	4.2	23	
PEG 8000	Spray Drying	Distilled water	25%	NR	101	4.4	2.7	
PEG 8000 laurate	Spray Drying	Distilled water	25%	NR	94.6	4.2	2.7	
PEG 8000 palmitate	Spray Drying	Distilled water	25%	NR	98.9 ± 2.6	4.4	2.5	
PEG 8000 behenate	Spray Drying	Distilled water	25%	Partial amorphous	99.1	4.4	2.8	
PEG 6000	Rotary Evaporation	HCl pH 1.2	25%	NR	70.23 ± 4.21	2.6	11.3	[21]
PEG 6000	Rotary Evaporation	Phosphate buffer pH 6.8	25%	NR	69.41 ± 5.29	2.4	13.8	
PEG 6000	Rotary Evaporation	HCl pH 1.2	33%	NR	64.24 ± 6.23	3.4	20	
PEG 6000	Rotary Evaporation	Phosphate buffer pH 6.8	33%	NR	58.79 ± 5.86	2.2	54	
PEG 6000	Rotary Evaporation	HCl pH 1.2	50%	NR	60.29 ± 5.22	2.9	39.5	
PEG 6000	Rotary Evaporation	Phosphate buffer pH 6.8	50%	NR	54.21 ± 5.71	2.1	83.6	
PEG 4000 palmitate	Freeze Drying	Distilled water	25%	Partial amorphous	73.6 ± 1.83	3.3	7.43	[6]
PEG 8000 palmitate	Freeze Drying	Distilled water	25%	Partial amorphous	98.38 ± 3.7	4.5	8.96	
Soluplus	Hot-Melt Extrusion	Phosphate buffer pH 6.8	12.5%	Amorphous	86.567	4.7	4.6	[17]
	Rotary Evaporation	Acidic buffer pH 1.2	50	NR	75.24 ± 2.63	2.6	5.4	[21]
	Rotary Evaporation	Acidic buffer pH 1.2	33	NR	78.96 ± 3.66	2.8	3.8	
	Rotary Evaporation	Acidic buffer pH 1.2	25	Amorphous	87.69 ± 4.12	3.1	3.8	
	Rotary Evaporation	Phosphate buffer pH 6.8	50	NR	64.78 ± 3.86	3.1	44.9	
	Rotary Evaporation	Phosphate buffer pH 6.8	33	NR	70.52 ± 5.01	3.4	30.1	
	Rotary Evaporation	Phosphate buffer pH 6.8	25	Amorphous	78.23 ± 4.11	3.8	10.5	
	Rotary Evaporation	Degassed water	10	Amorphous	71.58	1.8	4.63	[20]
	Hot-Melt Extrusion	Degassed water	10	Amorphous	90.78	2.3	3.38	
	Rotary Evaporation	Acidic buffer pH 1.2	10	Amorphous	69.48	1.8	4.31	
	Hot-Melt Extrusion	Acidic buffer pH 1.2	10	Amorphous	87.27	2.2	4.31	
	Rotary Evaporation	Phosphate buffer pH 6.8	10	Amorphous	82.86	2.1	4.31	
	Hot-Melt Extrusion	Phosphate buffer pH 6.8	10	Amorphous	89.74	2.3	3.38	
Polyvinyl pyrrolidone K30	Spray Drying	0.1 M HCl pH 1.2	20	Amorphous	97.2 ± 0.9	3.2	3.7	[5]
	Spray Drying	0.1 M HCl pH 1.2	25	Amorphous	93 ± 2.4	3.1	3.7	
	Spray drying	0.1 M HCl pH 1.2	33	Amorphous	78.6 ± 1.7	2.6	12.4	
Polyvinyl pyrrolidone K15	Rotary Evaporation	0.1 N HCl pH 1.2	50	NR	63.2 ± 8.6	1.8	22.54	[18]
	Rotary Evaporation	0.1 N HCl pH 1.2	25	NR	73.0 ± 8.3	2.0	3.48	
	Rotary Evaporation	0.1 N HCl pH 1.2	16.67	NR	72.4 ± 3.6	2.0	3.48	
	Rotary Evaporation	0.1 N HCl pH 1.2	12.5	NR	72.8 ± 5.1	2.0	3.48	
Polyvinyl pyrrolidone K30	Rotary Evaporation	0.1 N HCl pH 1.2	50	NR	66.2 ± 7.3	1.9	15.25	
	Rotary Evaporation	0.1 N HCl pH 1.2	25	NR	70.8 ± 4.3	2.0	3.63	
	Rotary Evaporation	0.1 N HCl pH 1.2	16.67	NR	77.5 ± 0.1	2.2	3.63	
	Rotary Evaporation	0.1 N HCl pH 1.2	12.5	Amorphous	77.8 ± 1.4	2.2	3.63	
Polyvinyl pyrrolidone K90	Rotary Evaporation	0.1 N HCl pH 1.2	50	NR	72.5 ± 5.1	2.0	7.71	
	Rotary Evaporation	0.1 N HCl pH 1.2	25	NR	72.2 ± 6.9	2.0	4.14	
	Rotary Evaporation	0.1 N HCl pH 1.2	16.67	NR	70.6 ± 4.4	2.0	4.14	
	Rotary Evaporation	0.1 N HCl pH 1.2	12.5	NR	71.5 ± 6.3	2.0	4.98	
Poly vinyl pyrrolidone Vinyl acetate 64	Hot-Melt Extrusion	pH 6.8 phosphate buffer	12.5	NR	60.59	3.3	20.6	[17]
	Hot-Melt Extrusion	0.1 N HCl pH 1.2	10	Amorphous	80.26	2.1	3.7	[20]
	Rotary Evaporation	0.1 N HCl pH 1.2	10	Amorphous	85.1	2.2	3.17	
	Hot-Melt extrusion	Degassed water	10	Amorphous	95.56	2.4	2.73	
	Rotary Evaporation	Degassed water	10	Amorphous	94.64	2.4	2.73	
	Hot-Melt Extrusion	Phosphate buffer pH 6.8	10	Amorphous	94.9	2.4	3.04	
	Rotary Evaporation	Phosphate buffer pH 6.8	10	Amorphous	93.98	2.4	2.73	
Hydroxypropylmethyl cellulose	Spray Drying	Water	85	NR	41.58	1.4	>30	[15]
	Spray Drying	Water	75	NR	54.1	1.9	>30	
	Spray drying	Water	50	NR	83.59	2.8	2.5	
	Spray drying	Water	15	Crystalline	86.67	2.9	2	
Silicon dioxide	Rotary Evaporation	Water + 0.2% SDS	67	NR	57.732	1.1	51.2	[23]
	Rotary Evaporation	Water + 0.2% SDS	50	NR	59.536	1.2	43.8	
	Rotary Evaporation	Water + 0.2% SDS	33	NR	69.845	1.4	33.8	
	Rotary Evaporation	Water + 0.2% SDS	20	NR	74.227	1.5	18.8	
	Rotary Evaporation	Water + 0.2% SDS	11.11	Partial Amorphous	85.825	1.7	4.8	
Chitosan	Spray Drying	Phosphate buffer pH 7.0	25	Partial Amorphous	74.818	1.4	4	[22]
	Spray Drying	Phosphate buffer pH 7.0	20	Partial Amorphous	76.642	1.5	13	
	Spray Drying	Phosphate buffer pH 7.0	16.67	Partial Amorphous	79.197	1.5	13	
Poloxamer 188	Hot-Melt Extrusion	pH 6.8 phosphate buffer	12.5	NR	18.5	1.0	>120	[17]

**Abbreviations:** NR: not reported.

**Table 3:** In vivo studies of andrographolide solid dispersion.

Polymer Matrices	Study Design	Type of Animal	Sex	Weight	No. of Subjects	C_max_	T_max_ (h)	AUC (µg/Lh)	AUC Improvement (Fold)	References
Polyethylene glycol 6000	Single dose	Wister rats	Male	200–250 g	30	0.91 ± 0.13 (mg/L)	1.03 ± 0.25	3.36 ± 0.23 (mg/Lh)	1.23	[17]
Soluplus	Single dose	Wister rats	Male	200–250 g	30	2.91 ± 0.12 (mg/L)	1.02 ± 0.11	8.72 ± 1.36 (mg/Lh)	3.2	[17]
	Single dose	Albino rats	Both	250–300 g	18	35.22 ± 3.54 (µg/L)	1	99 ± 11.45 (µg/Lh)	1.18	[16]
PVP K30 and polyethoxylated castor oil	Single dose	Sprague Dawley rats	Male	200 g	10	254.0 ± 59.7 (ng/mL)	0.4 ± 0.3	928.2 181.1 (ng h/mL)	0.99	[18]
HPMC	Single dose, randomized, two-way crossover	Beagle dogs	NA	10 kg	6	87.54 ± 54.82 (µg/L)	1.38 ± 0.41	495.86 ± 281.05 (µg/Lh)	N/A	[10]
	Single dose, randomize	Wistar rat	Male	200 g	18	346.741 ± 38.163 (µg/L)	0.36 ± 0.17	1564.784 ± 416.853 (µg/Lh)	4.12	[15]
HPMC with lactose, and sodium carboxymethyl starch	Single dose, randomize	Wistar rat	Male	200 g	18	323.423 ± 43.527 (µg/L)	0.44 ± 0.14	1794.738 ± 311.213 (µg/Lh)	4.73	[15]
PVPVA 64	Single dose	Wister rats	Male	200–250 g	30	1.59 ± 0.32 (mg/L)	1.13 ± 0.68	4.76 ± 0.28 (mg/Lh)	1.75	[17]
Poloxamer 188	Single dose	Wister rats	Male	200–250 g	30	0.96 ± 0.26 (mg/L)	1.23 ± 0.24	3.41 ± 0.36 (mg/Lh)	1.25	[17]

## Data Availability

Data used in this study are available upon appropriate request.

## Supplementary Materials

The following supporting information can be downloaded at https://www.mdpi.com/article/10.3390/pharmaceutics16050688/s1, Table S1. Search terms.
