# Supplementary material for: Polymer Matrix and Manufacturing Methods in Solid Dispersion System for Enhancing Andrographolide Solubility and Absorption: A Systematic Review
Source: Pharmaceutics. 2024 May 20;16(5):688. doi: 10.3390/pharmaceutics16050688 (PMC11125128; doi:10.3390/pharmaceutics16050688)
Supplement: Supplementary file 1 [file pharmaceutics-16-00688-s001.zip › pharmaceutics-2971026-supplementary.pdf]

Table S1. Search terms

Terms for andrographolide		Terms for solid dispersion
andrographo*		solid dispersion
"Andrographis paniculata"[Mesh]		amorphous dispersion
"Andrographis"[Mesh]	AND	amorphous solid dispersion
andrographolide		
'andrographolide'/exp		
'andrographis'/exp		

Note: We combined within terms by OR and between terms by AND
